# Feasibility and acceptability of home delivery of water for dental caries control in Latinx children—“Sediento por una Sonrisa,” Thirsty for a Smile: Single-arm feasibility study

**DOI:** 10.3389/fpubh.2022.916260

**Published:** 2022-09-20

**Authors:** Joana Cunha-Cruz, Linda K. Ko, Lloyd Mancl, Marilynn L. Rothen, Catherine Harter, Juliana B. Hilgert, Mark K. Koday, Stephen Davis

**Affiliations:** ^1^Department of Clinical and Community Sciences, School of Dentistry, University of Alabama at Birmingham, Birmingham, AL, United States; ^2^Department of Health Systems and Population Health, School of Public Health, University of Washington, Seattle, WA, United States; ^3^Department of Oral Health Sciences, School of Dentistry, University of Washington, Seattle, WA, United States; ^4^Department of Assessment, Planning and Development, Tacoma Pierce County Health Department, Tacoma, WA, United States; ^5^Department of Preventive and Social Dentistry, Dental School, Universidade Federal do Rio Grande do Sul, Porto Alegre, RS, Brazil; ^6^Yakima Valley Farm Workers Clinic, Yakima, WA, United States

**Keywords:** dental caries, behavioral intervention, environmental restructuring, practice-based research (PBR), sugar consumption, nutrition, oral health, sugar sweetened beverages

## Abstract

**Background:**

Outcomes of surgical treatments under general anesthesia for early childhood caries of young children from low-income groups are poor requiring retreatment within 2 years. Dietary sugar is an ideal intervention target given that it is the most prominent risk factor for dental caries and there is increasing evidence of successful interventions to reduce its intake. Our aim is to investigate the feasibility and acceptability of the Thirsty for a Smile intervention, designed to promote consumption of water in lieu of sugar sweetened beverages, among children who underwent surgery for early childhood caries and their caregivers, mostly from Latino heritage.

**Methods:**

A single-arm feasibility study was conducted in a dental practice from a community health center in eastern Washington State. Bottled water was delivered to the participants' homes and caregivers received patient-centered counseling for setting goals to increase children's water intake and reduce sugar sweetened beverages consumption. We assessed the feasibility and acceptability of the intervention and study procedures through participation rates, interviews and a questionnaire completed by the caregivers. Data was analyzed and themes and descriptive statistics presented.

**Results:**

Twenty-two dyads of caregivers and their children between 2 and 9 years old who recently had surgical treatment for early childhood dental caries were enrolled. All study assessments were completed by more than 90% of participants, except for the final 24-h dietary recall (73%). Dietary counseling, both in person and brief telephone calls, was highly acceptable to the caregivers, and they also reported their children enjoyed and used the water bottles. On a scale from 1 to 10, the average rating for the helpfulness of the dietary counseling component for changing child's drinking habits was 9.62 and for the water delivery component, 8.86.

**Conclusions:**

This study tested the feasibility of conducting a trial in a dental practice setting, and the acceptability among caregivers of young children who underwent surgery for early childhood caries. It demonstrated that the Thirsty for a Smile intervention and study processes were feasible and acceptable. The study provides useful information for implementation of a two-arm randomized controlled trial in this setting and may also benefit other researchers attempting to test similar interventions.

## Introduction

Dental caries is a multifactorial, non-communicable disease that develops when bacteria in the mouth metabolize sugars to produce acid that demineralizes the hard tissues of the teeth (enamel and dentine) (1). Untreated caries in permanent teeth affects 2.3 billion people worldwide and in deciduous teeth affects 532 million children (2). These unmet treatment demands have significant public health implications (3). Latinx children have some of the highest rates of early childhood caries in the United States and are less likely than white children to receive necessary treatment (4, 5). The prevalence of untreated decay in children is about 17% for all children in the US, and 21% for Hispanic and Latinx children (6). With limited access to dental care, there is an even higher risk for developing dental caries for those children living in low-income or rural areas (7, 8).

To treat children with severe early childhood caries, general anesthesia is often required to restore and remove affected teeth due to the inability of children to cooperate and the severe, and generalized nature of this condition (9). Treatment of dental caries under general anesthesia (GA) is becoming more common and costly (10) and has an emotional and financial impact on the family (11). Caregivers of children undergoing this treatment reported that the surgical events elicit guilt, anxiety, and a sense of own accountability for development of severe caries in their children (11). More importantly, the outcome of this treatment is poor: 37–79% of treated children develop new carious lesions within 24 months (12–16), and 17% require surgical retreatment within 2 years (16). A comprehensive approach that targets the main causes of dental caries is necessary to improve the clinical outcomes for severe early childhood caries (17). Dietary sugar seems to be an ideal intervention target given that it is the most prominent risk factor for dental caries (18, 19) and there is increasing evidence of successful interventions to reduce its intake (20–23). Sugar sweetened beverages (SSBs) represent about 50% of all added sugar consumption by children (24) and contribute significantly to children consuming nearly double the recommend amount of <10% of total calories (25) and their intake is associated with early childhood caries (26, 27). Trials of one-on-one dietary counseling undertaken in dental settings have not been promising (28, 29), but trials of restriction of SSBs for reducing other chronic diseases (e.g., obesity) have been successfully implemented (20, 21) and can be adapted to clinical settings. Evidence is mounting that interventions to reduce SSBs (20–23), including interventions promoting water consumption to displace SSBs (30), can be effective.

An evidence-based theoretically informed intervention was designed to support dietary changes of children after dental treatment under general anesthesia. To ensure a successful randomized controlled trial, it is recommended that pilot and feasibility studies are first conducted (31, 32) and acceptability of the intervention assessed (33). Eldridge et al. developed a conceptual framework defining feasibility and pilot studies (34) as: “A feasibility study asks whether something can be done, should we proceed with it, and if so, how. A pilot study asks the same questions but also has a specific design feature: a pilot study for a future study, or part of a future study, is conducted on a smaller scale”. Outcomes of feasibility studies are related to the ability to conduct the study intervention and measurements (31). Acceptability has been defined as a “multifaceted construct that reflects the extent to which people delivering or receiving a healthcare intervention consider it to be appropriate, based on anticipated or experienced cognitive and emotional responses to the intervention” (33) and assessing it helps to identify any modifications required that may increase the likelihood of participation in a trial, as well as the sustainability of the intervention (33). It can be measured quantitatively, by validated acceptability and satisfaction measures, and qualitatively, by asking open-ended pointed questions on how recipients of the intervention are interacting with it (33). This study aimed to investigate the feasibility and acceptability of the Thirsty for a Smile intervention, designed to promote consumption of water in lieu of sugar sweetened beverages, among children and their caregivers, mostly from Latino heritage.

## Methods

### Study design and setting

A single-arm feasibility study was conducted from March until December 2019. This study received ethics approval from the Institutional Review Board of the University of Washington. This project did not meet the National Institutes of Health (NIH) definition of a clinical trial as it did not study the cause-and-effect relationship between an intervention and a health outcome. Therefore, this study was not registered in a public trials' registry such as ClinicalTrials.gov.

The study settings were two dental clinics located in a Community Health Center. The health center provides medical, behavioral, and dental care to mostly rural communities in eastern Washington State, United States. Half the population in the rural communities are Hispanic or Latino and about 1/3 (38%) speak a language other than English at home. In 2020, the health center served more than 170,000 patients: 44% were children, 65% were Hispanic/Latino, and 56% were at or below the federal poverty level. Approximately 700 children per year are treated under general anesthesia for early childhood caries in the two dental practices.

### Recruitment and participants

Participants were recruited at the clinical sites. The inclusion criteria were a child of age 2 to <9 years old, with a diagnosis of early childhood caries, with previous treatment under general anesthesia, and in good general health by parent report. The exclusion criteria were a child with American Society of Anesthesiologists' (ASA) physical status IV or higher (due to the potential that the child would not be able to drink the beverage independently) and parent/caregiver under 18 years of age. Caregivers of children meeting the inclusion criteria were invited to participate during their child's surgery visit or the surgery follow-up visit. Prior to data collection a consent form describing in detail the study procedures and risks was given to the caregivers and a research coordinator explained the study and answered questions. Informed consent was then obtained from caregivers and assent was obtained from children 7 years of age and older. Both forms were available in English and Spanish, and the consent process was conducted by bilingual research coordinators.

### Study intervention

The intervention had an environmental component and a behavioral component. The environmental component involved altering the home environment by providing bottled water. Water bottles were used because many Hispanic and Latino families distrust tap water safety (35, 36). Child friendly, brightly colored, animal shaped plastic bottles with re-sealable spouts containing water with ~0.7 parts per million (ppm) of fluoride were delivered to the participants' home every other week for 2 months. The behavioral component consisted of counseling sessions delivered by a trained dietitian to help parents set goals and an action plan to overcome barriers to decreasing their children's consumption of sugar sweetened beverages and increasing their consumption of water (37). Caregivers participated in two in person sessions: one at baseline and one two months after baseline. They also participated in 4 telephone check ins between the baseline and final visits. The dietitian interventionist was trained to defined performance criteria through roleplaying exercises which were scored according to pre-established fidelity checklists of the essential elements necessary in the sessions (e.g., assessing motivation, goal setting, and anticipating challenges).

### Procedures

#### Schedule

**Figure 1:**
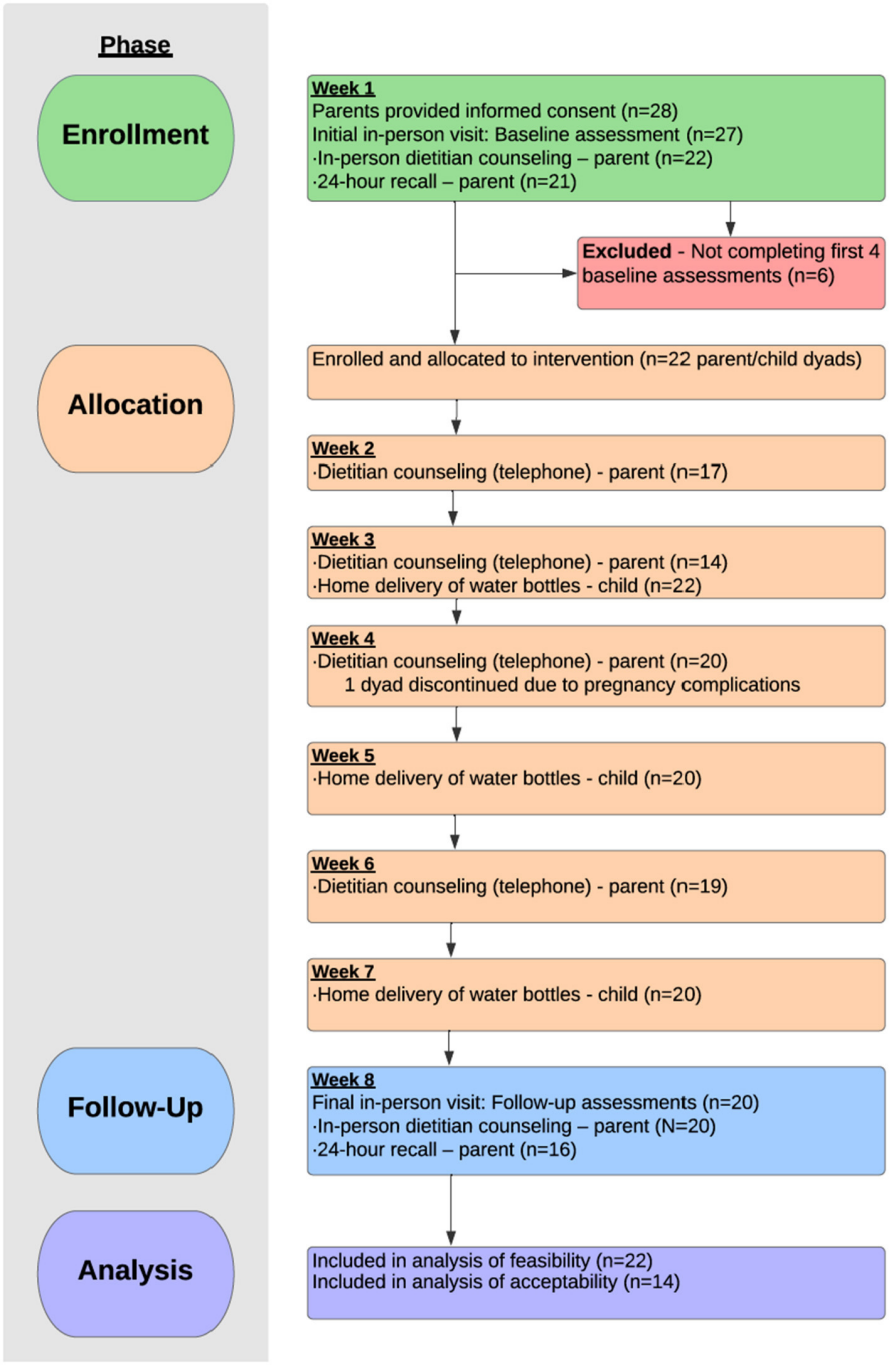
Flow of participants through the stages of the Thirsty for a Smile study.

The study baseline visit was conducted during the child's follow-up recall after dental surgery. Dental caries experience was recorded using the International Caries Detection and Assessment System (ICDAS) and height, weight and waist circumference measurements were obtained from the child and caregiver. The caregiver completed the baseline questionnaire using a tablet. The behavioral counseling was scheduled with the dietitian conducting the intervention within 2–3 days of the baseline dental exam. At the dietitian's visit, the caregiver received the first 2-week supply of water bottles. The dietitian informed the caregiver that they would be contacted by telephone the following week for a 24-h dietary recall interview of about 45 min duration. The calls were not scheduled in advance purposefully and the interviewer asked caregivers to list in sequence all foods and beverages consumed by the child during the previous day, and then asked the caregiver to provide details (e.g., portion sizes, brand names, preparation, or cooking methods). Over the two-month study period, telephone check-ins were conducted, and home water delivery took place every 2 weeks. The caregiver and child returned for the final study visit at 2 months for recording of dental caries and anthropometric measurements, completion of questionnaire and the final in-person visit with the dietary interventionist. This was followed by a second 24-h dietary recall interview (Figure 1). At the end of the intervention period for all participants, semi-structured group and individual interviews were conducted.

#### Data collection

To assess the feasibility and acceptability of the intervention and study procedures, both quantitative and qualitative data were obtained. Data on participation in the different intervention components and study assessments were obtained from forms created in REDCap (Research Electronic Data Capture) to track the study activities for each participant dyad. Data tracked included the water deliveries, the in-person and telephone behavioral counseling sessions, the baseline and final examination for dental caries and anthropometric measures, the caregiver questionnaire, and the telephone dietary recall interviews. Guided by recommendations of the Treatment Fidelity Workgroup of the NIH Behavior Change Consortium (80), fidelity of the intervention was monitored and rated. For each type of session (first and final in-person visits and telephone check-ins) a behavioral trainer listened to the 25% or more of the audio recordings, evaluated fidelity using checklists, scored the interventionist competency, and then provided feedback to the dietitian. The competency ratings used to score the essential elements of each session were 1 (poor), 2 (adequate) and 3 (exemplary).

Data on acceptability of the intervention components, and of data collection methods were obtained through the questionnaire completed by the caregivers and through semi-structured group and individual interviews at the end of the intervention period. After the intervention, caregivers were asked to rate on a scale from 1 to 10 how helpful or enjoyable the intervention components were and how likely they would recommend the study to a friend or relative (see **Table 2**). Using a 5-point Likert scale, caregivers were also asked, at the end of the questionnaire both at baseline and final visits, about ease of use, satisfaction, helpfulness, and completion time of the questionnaire (28). A bilingual bicultural research staff conducted the in-person semi-structured Spanish interviews at the end of the intervention period at the dental clinic. All participants of the study were invited to the interview session and celebration of the end of the study. The session was scheduled on a weekend day and childcare was provided. The interviewer followed a guide with open-ended questions developed by the investigators to elicit participants' perceptions of the study including the coordination and intervention components as well as their recommendations for improving the study. The group interview lasted 70 min and the individual interview, 30 min. The individual interview was conducted because the participant caregiver was available for the interview at a different time than the rest of the group.

Additional details of the study design and data collection are available in the published protocol, including the clinical and behavioral outcome data collected related to dietary behaviors, oral health, and weight (37).

#### Data analysis

The sample size was based on the estimated number of child-caregiver dyads that could be recruited from among the number of children typically scheduled for S-ECC treatment of dental caries under general anesthesia in a 4-month period. Feasibility outcomes were measured by completeness of trial outcome data collected, behavioral session attendance, adherence to water deliveries, and study retention. Acceptability outcomes were measured quantitatively, by validated acceptability and satisfaction measures (38), and qualitatively, by asking open-ended pointed questions on how recipients of the intervention were interacting with it.

Interview recordings in Spanish were transcribed verbatim, then translated to English, and checked for accuracy. Two members of the research team (CH, JH) conducted coding and thematic analysis. Coding was an iterative process where the transcripts were first reviewed, then the coders independently coded the transcripts and met to discuss their agreements and discrepancies, until the codes were reconciled and combined into candidate themes. Code reports were produced and then synthesized into theme domains and subdomains with associated quotes, forming the basis of the findings (39).

## Results

### Participants

Recruitment took place over seven months. Of the 94 children who met the eligibility criteria for participation, 37% of caregivers expressed interest in study participation. Twenty-two children and their caregivers (23%) enrolled in the study and 20 completed the study (91% completion rate). Among the 22 children enrolled in the study, 77% were from Hispanic or Latino(a) ethnicity, mostly from Mexican or Mexican American descent, and their median age was 5, ranging from 3 to 8 years old (Table 1). All caregivers were women, except one. Twenty of the caregivers were the mother, one was the child's father, and one was the child's grandmother. Their median age was 31 years old, and 36% had completed high school. The questionnaire at the end of the intervention period was completed by 19 caregivers (86%), and 14 (64%) completed the last section of this questionnaire related to acceptability of the data collection method through the questionnaire in the tablet. Five caregivers participated in the group interview and 1 in an individual interview (27%).

**Table 1:** Characteristics of the participants of the Thirsty for a Smile study.

	**Child**	**Caregiver**
**Age (years)**		
Median	5	31
Minimum – maximum	3–8	21–45
**Gender**		
Female	11	21
Male	11	1
**Hispanic/Latinx ethnicity**		
Yes	17	18
No	3	3
Unknown	2	1

### Feasibility and acceptability of assessments

All study assessments were completed by more than 90% of participants, except for the final 24-h dietary recall (73%). At the baseline data collection, 99% of the assessments were completed and at final follow-up data collection, 85% of the assessments were completed. The completion rate at baseline was 100% for the dental caries and anthropometric measurements, and 95% for the 24-h dietary recall and the questionnaire. At the final follow-up, 91% completed the dental caries and anthropometric measurements, 73% the 24-h dietary recall, and 86% the questionnaire. The lower completion rate for the final 24-h recall was due to being unable to schedule the caregiver for the dietary recall after five attempts to contact. The lower response rate for the questionnaire at the final data collection may be due to the length of the questionnaire, particularly for those participants who answered it in Spanish.

A lower percentage of caregivers at the final data collection indicated that they were satisfied with the survey (76%), and that the amount of time to complete the questionnaire was somewhat or very acceptable (62%) than at the baseline data collection (86 and 77%, respectively). The mean completion time was 29 min (±10) at baseline and 26 min (±8) at final data collection. Most participants found that the REDCap survey tool in a tablet was easy to use (59%), with about half (45.5%) of participants needing help at baseline and only 19% at final data collection. The majority of the participants indicated that the questionnaire was helpful in describing their child's experience of oral health and diet (86% at baseline and 71% at final data collection).

### Feasibility and acceptability of the environmental component: Home delivery of water bottles

For the environmental component, all study participants received the four water bottle deliveries, except for two participants who elected not to receive the last two deliveries. One discontinued water delivery because the child did not like the taste of the water, and the other participant dropped out of the study due to the caregiver's health issues.

Caregivers reported their children enjoyed and used the water bottles. On a scale from 1 to 10, the average rating for the helpfulness of the water delivery component was 8.86. The average rating for the child's acceptability of the water bottles was 9.57 (Table 2).

**Table 2:** Acceptability and helpfulness of the Thirsty for a Smile program as rated by the participants.

	**Mean (SD)**
How much did *the water deliveries* help you make changes so your child would drink more water and fewer sugary drinks like soda and prepackaged juices?	8.86 (±2.41)
In general, how much did your child *enjoy the animal-shaped water bottles*?	9.57 (±0.85)
How much did *the conversations with the dietitian* help you make changes so your child would drink more water and fewer sugary drinks such as soda and prepackaged juices?	9.62 (±0.65)
What did you think about the *number of phone calls* you received from the dietitian?	about right = 100%
*Overall*, how much did the program help your child drink more water and fewer sugary drinks like soda and prepackaged juices?	9.38 (±1.12)
How likely is it that you would recommend the Thirsty for a Smile program to a friend or relative?	9.64 (±0.84)

During the interviews, caregivers discussed how they and their kids liked getting bottles delivered to their homes. For the kids, they said it brought excitement to anticipate drinking the “special” water. For the parents, it removed one extra step to get their kids to drink more water instead of sugary drinks.

“*It was important because it was harder to go pick them up somewhere, to have them when they were running out. She (my daughter) already knew— “my waters will arrive soon. I'll have to drink that water.” If they hadn't arrived, she would have said, “well it's over,” but no. It was important [to receive the bottles at home], and they did it well.”*

When it came to using the water bottle, all participants described positive experiences. Multiple participants talked about taking the bottles on trips where their child would ask for a beverage, and they would be able to give them the water as a substitute because they had it on-hand.

“*On every occasion, we'd take a box. We would go to a store, and she'd say, “I want juice.” I would tell her, “No, you have water.” “Okay, then.” She already knew she had to take one or nothing, so she did”*

Another participant described how easy it was to fill her purse or bag with a bottle or two, describing the size and shape of the bottles themselves as easy to transport.

“*I started bringing the bottles with me, I could tell him, “Oh no, look I brought this for you,” and just because of the color and the little animal; he'd just sit still there with his water instead of wanting a juice or something from the store, and every time we'd go to the park or go anywhere, I had some bottles in my bag.”*

Others mentioned how the children knew the boxes of water bottles were there for them, so eventually some started to take the bottles themselves, giving some autonomy to the children about their choices. Some described instances in which other children in the household started to drink the water bottles as well.

### Feasibility and acceptability of the behavioral component: Dietitian counseling

For the behavioral component, all caregivers received in-person counseling sessions with the dietitian at baseline and 20 caregivers received it at final study visit. Ten caregivers completed all 4 telephone check-ins with the dietitian, 8 completed 3 check-ins, and the remaining 4 completed <3 check-ins. Fidelity of the intervention was very good with an average fidelity score of 1.97 for the first in-person dietitian visit, 1.98 for the telephone check-ins, and 1.92 for the final in-person visit (rated on a 2-point scale).

The behavioral dietary counseling, both in person and brief telephone calls, was highly acceptable to the caregivers. On a scale from 1 to 10, the average rating for the helpfulness of the dietary counseling component for changing child's drinking habits was 9.62. All caregivers reported that the number of phone calls with the dietitian were about right (Table 1).

During the interviews, caregivers also mentioned that the duration of the phone calls was acceptable. Others mentioned how if they could not speak when the dietitian called at first, it was easy to reschedule for a different time.

“*Everything was perfect. If you couldn't talk, they'd return the call when you were available. They always found a way to get your information when you weren't busy. When there was a need for them to be longer, they were. When it wasn't necessary because we were doing well, they were short.”*

### Overall acceptability

The high participation rates in the assessments and intervention components indicated that the program was acceptable to the children and caregivers. Caregivers also rated the overall helpfulness of the program in helping their child drink more water and fewer sugary drinks like soda and prepackaged juices a 9.38, on a scale from 0 to 10. They also would highly recommend the program to a friend or relative (9.64). During the interviews, participants described the ways in which they grew and learned new things about themselves as well as their families. One participant described these new habits not just as simply swapping one drink for another for their child, but also changing their mindset and how they decide what to eat as a family.

“*It's not just drinking water and not eat sugary things. It's more about, “how are we eating? Are we teaching her right or not?” It turned from one small thing to another big thing, it spread. We applied this to other things at home, so we've learned a lot. I even started losing weight.”*

Some described that they now understood their role in their child's health and diet and took accountability for their choices.

“*More than anything, we control what they do or take. If we just give up when they want juice, or soda—it's our fault, not theirs. —It's not like they can go buy it, we do. We provide it for them, so we're mostly to blame for what they consume. That's something I learned here.”*

One participant noted how this intervention changed her views, and her child's views on oral health and its importance for overall health.

“*I learned that teeth are also a big part of our health. I learned how water helps us with our teeth, how it helps our health, that teeth aid our health. She [my daughter] learned a lot as well because she learned the importance of her teeth.”*

## Discussion

This study demonstrated that a combined environmental and behavioral intervention is feasible and perceived as highly acceptable by caregivers and children post-surgery for early childhood caries. The proposed Thirsty for a Smile intervention trial was evaluated in this pilot study to determine the potential to enroll and retain participants in a longer efficacy trial. Trials that are feasible to conduct and acceptable to participants are considered to have an increased likelihood of reaching sample size and retention goals.

### Feasibility: Can we do it and how?

Our outcome measures of feasibility demonstrated that the study as conceived can be conducted. The enrollment goal of 21 child-caregiver dyads was exceeded as was the goal to retain >90% of the participants. The feasibility of the environmental component-95.5% home delivery of child friendly water bottles intended to lower the resources barrier—exceeded our goal of ≥90%. The fidelity of the behavioral component (counseling designed to strengthen caregiver self-efficacy for implementing change in the child's diet) was high and our goals for baseline (100%) and follow up participation (≥90%) for in-person dietitian visits were met. However, the weekly phone calls fell short. Attendance at the in-person dental/physical exams was the same as the in-person dietitian visits meeting our goals. The dietary recall assessments which were conducted over the phone fell slightly short of our goal at baseline and somewhat shorter at follow up though more than 2/3 of caregivers still responded. It is interesting that on closer examination, the in-person visits had >90% follow up, while the phone interventions were less successful. This enthusiasm for in-person participation is particularly encouraging in a population primarily of farm workers for a study conducted from spring to autumn.

Meeting these goals was not without challenges. Feasibility assessments and adjustments were made in real-time throughout the study. Recruitment posed the first challenge when it became apparent that the goal of recruiting 21 child-caregiver dyads would not be met in 4 months. The study coordinator position required more time than anticipated during the recruitment phase as the need to be flexible and available in the clinic most days was necessary for effective recruitment. When additional time was allotted and an outreach coordinator with the required communication skill set and flexible hours was identified for the role, recruitment went from 4 dyads enrolled in 3 months to 18 enrolled in the next four months. The environmental component of water delivery on time was challenging when participants unexpectedly changed residences, which occurred on several occasions. Problem solving included finding the means for delivery to the new address or caregiver pick up of water bottles in a timely manner and coordinators learned that regular contact with caregivers to learn about new addresses and to determine best means of delivery or pickup was important. An additional consideration is for caregivers who are pregnant as the pregnancy can affect the caregivers' participation and therefore the child's participation in the study. Flexibility to accommodate needs and schedules can keep these interested caregivers and children participating in the study.

### Acceptability: Do participants consider it appropriate or are modifications needed?

We were able to determine which parts of the intervention were accepted as is, which could use improvements, and that no portions were unacceptable as to require them to be removed or reimagined. Caregivers reported that they approved of the technology required to complete surveys and the amount of time to complete them. Additionally, caregivers that participated in the group interviews described the dietitian visits and calls as appropriate in length of time and easy to participate. They acknowledged the adverse effects of sugary drinks on young children's oral health and rated high conversations with the dietitian in helping them make changes to reduce sugary drink consumption. All caregivers liked receiving the water bottles to their homes instead of having to pick them up. When asked about using the water bottles, caregivers mostly described positive experiences. Kids responded well to the bottles and over time they facilitated in the children choosing water independently over sugar sweetened beverages.

No modifications to address acceptability were made during the study as acceptability assessments were made at the conclusion of the study through the final survey and the interviews. However, despite reports of very good acceptability, consideration can be given to improving several aspects such as the acceptance of the oral health questionnaire administered at the final follow-up visit. The timing, length or mode of completion are areas to evaluate. Reaching caregivers by phone was sometimes challenging. If the phone is ever present, another method of contact such as a text to schedule a phone call or to conduct a check-in could be appropriate. The 24-h dietary recall at the final follow up visit was the least completed component of study data collection. The person conducting the calls made five attempts on different days of the week or times of day to reach the caregiver. Alternate contacts were also called. It may be that caregivers considered participation in the study complete following the final visit with the dietitian and though the dietitian reminded them of the 24-h recall, it may no longer have been salient. Conducting the dietary recall prior to the last in-person visit or providing an incentive for completing it may improve response.

### Strengths and limitations of the study

Our study achieved its aims, but as a feasibility study, our sample size was small, and the length of the study was short; enrollment was limited to 22 child-caregiver dyads, and they were enrolled in a period of 7 months for a total study length of 9 months from first enrollment to last follow-up visit. Our findings should not be generalized to other populations. For example, our intervention might not be acceptable to caregivers whose children do not require surgery to treat dental caries because of a lower level of disease combined with the child's ability to tolerate routine dental care. It also may not be applicable to populations who do not have similar distrust of tap water. The children in this study aged between 3 and 8 years old, and although this intervention may prove effective for children beyond that age range, our focus was early childhood caries only and did not extend to children with full permanent dentition, nor to children old enough to make independent decisions about their drink choices and who experience a greater sphere of social influences. Lastly, data was collected through self-reports from caregivers and they were interviewed by the academic research team, and assisted by the on-site research team member when completing the oral health questionnaire, and not all caregivers participated in these activities. This sample of participants may not provide the full range of opinions related to the study.

## Conclusions

Several interventions have been evaluated for prevention of early childhood dental caries including changes in oral hygiene habits, oral health education, and fluoride treatments (40, 41). To prevent early childhood dental caries relapse, our intervention focused on reducing the consumption of sugar sweetened beverages by combining two strategies—a behavioral component and an environmental component: a dietitian was trained to provide counseling at the dental clinic and water bottles were home delivered. Others have used dental professionals to deliver dietary counseling to caregivers—dentists (42), dental hygienists (43) or dental teams (41) at similar settings. Similar to our study, these studies posit the dental office as an appropriate setting to implement this type of program. The role of the environment as a driver of consumption of sugar sweetened beverages is acknowledged in other studies and emphasized by health organizations and policy makers (44). Most environmental interventions to increase water consumption to decrease sugar sweetened beverage consumption in children did so in schools or recreation centers, but for young children as the ones with early childhood caries the home environment is a more appropriate setting to intervene. Home-based interventions seem to be more effective in increasing water consumption and reducing weight (44, 45), but evidence from randomized controlled trials for reducing dental caries is lacking (45).

Our study assessed feasibility and acceptability of the Thirsty for a Smile intervention and study procedures. A future trial would address the efficacy of the intervention with a larger sample. If the intervention is proven efficacious, a multicenter trial could test effectiveness of the intervention. With demonstrated effectiveness, this comprehensive treatment approach that targets the cause of the disease could be covered by health insurance providers on a time-limited basis to be implemented by health care organizations, thus preventing recurrent disease, hospitalization, and general anesthesia of young children. Our study suggests that provision of fluoridated drinking water to the home in child-friendly water bottles coupled with behavioral counseling is feasible in this dental practice setting as well as acceptable to caregiver-child dyads.

## Data availability statement

The datasets presented in this article are not readily available because informed consent did not include a statement that the anonymized data would be publicly available. Requests to access the datasets should be directed to JC-C, JoanaCCruz@uab.edu.

## Ethics statement

The studies involving human participants were reviewed and approved by Institutional Review Board of the University of Washington. Written informed consent to participate in this study was provided by the participants' legal guardian/next of kin.

## Author contributions

JC-C led the overall study. CH, JH, and LM conducted the analysis. JC-C, CH, JH, LM, and MR drafted the manuscript with input from the other authors. All authors contributed to the concept and design of the study, the interpretation of data, revision of the manuscript for important intellectual content, and have read and approved the final version of the manuscript.

## Funding

The research presented in this manuscript was funded by the US National Institutes of Health (NIH)—National Institute of Dental and Craniofacial Research (NIDCR) (R56DE027026). JH was funded for a sabbatical leave at the University of Washington by a PRINT grant from CAPES, Brazil (88887.363233/2019-00). The authors acknowledge support from the University of Washington Institute of Translational Health Sciences (ITHS) supported by grants UL1 TR002319, KL2 TR002317, and TL1 TR002318 from the NIH National Center for Advancing Translational Sciences through the Clinical and Translational Science Awards Program (CTSA).

## Conflict of interest

The authors declare that the research was conducted in the absence of any commercial or financial relationships that could be construed as a potential conflict of interest.

## Publisher's note

All claims expressed in this article are solely those of the authors and do not necessarily represent those of their affiliated organizations, or those of the publisher, the editors and the reviewers. Any product that may be evaluated in this article, or claim that may be made by its manufacturer, is not guaranteed or endorsed by the publisher.
